# Diffusion-weighted magnetic resonance imaging for differentiating between benign and malignant thoracic lymph nodes: a meta-analysis

**DOI:** 10.1590/0100-3984.2020.0084

**Published:** 2021

**Authors:** Francisco de Souza Santos, Nupur Verma, Guilherme Watte, Edson Marchiori, Tan-Lucien H. Mohammed, Tássia Machado Medeiros, Bruno Hochhegger

**Affiliations:** 1 Graduate Program in Internal Medicine and Health Sciences, Pontifícia Universidade Católica do Rio Grande do Sul (PUCRS), Porto Alegre, RS, Brazil.; 2 Department of Radiology, University of Florida (UF), Gainesville, FL, USA.; 3 Department of Radiology, Universidade Federal do Rio de Janeiro (UFRJ), Rio de Janeiro, RJ, Brazil.

**Keywords:** Diffusion magnetic resonance imaging, Lymphadenopathy, Lymph nodes/diagnostic imaging, Thoracic neoplasms/diagnosis, Magnetic resonance imaging, Meta-analysis, Difusão por ressonância magnética, Linfadenopatia, Linfonodos/diagnóstico por imagem, Neoplasias torácicas/diagnóstico, Ressonância magnética, Metanálise

## Abstract

**Objective:**

To establish the diagnostic performance of diffusion-weighted magnetic resonance imaging (DWI) in discriminating malignant from non-malignant thoracic lymph nodes.

**Materials and Methods:**

This was a meta-analysis involving systematic searches of the MEDLINE, EMBASE, and Web of Science databases up through April 2020. Studies reporting thoracic DWI and lymph node evaluation were included. The pooled sensitivity, specificity, diagnostic odds ratio, positive predictive value, negative predictive value, and area under the receiver operating characteristic curve (AUC) were calculated.

**Results:**

We evaluated six studies, involving a collective total of 356 mediastinal lymph nodes in 214 patients. Thoracic DWI had a pooled sensitivity and specificity of 92% (95% confidence interval [95% CI]: 71-98%) and 93% (95% CI: 79-98%), respectively. The positive and negative likelihood ratios were 13.2 (95% CI: 4.0-43.8) and 0.09 (95% CI: 0.02-0.36), respectively. The diagnostic odds ratio was 149 (95% CI: 18-1,243), and the AUC was 0.97 (95% CI: 0.95-0.98).

**Conclusion:**

DWI is a reproducible technique and has demonstrated high accuracy for differentiating between malignant and benign states in thoracic lymph nodes.

## Abstract (pt)

**Objetivo:**

Uma meta-análise foi realizada para estabelecer o desempenho diagnóstico da ressonância magnética com imagem ponderada em difusão (DWI) na discriminação de linfonodos torácicos malignos de benignos.

**Materiais e Métodos:**

MEDLINE, EMBASE e Web of Science foram sistematicamente pesquisados até abril de 2020. Foram incluídos estudos que relatavam o uso de DWI na avaliação de linfonodos torácicos. Sensibilidade, especificidade, razão de chances de diagnóstico, valores preditivos positivos e negativos e área sob a curva (AUC) foram calculados.

**Resultados:**

Foram encontrados 356 linfonodos mediastinais de 214 pacientes nos seis estudos incluídos. DWI produziu sensibilidade e especificidade combinadas de 92% (intervalo de confiança 95% [IC 95%]: 71-98%) e 93% (IC 95%: 79-98%), respectivamente. A razão de verossimilhança positiva foi de 13,2 (IC 95%: 4,0-43,8), a razão de verossimilhança negativa foi de 0,09 (IC 95%: 0,02-0,36); A razão de chances de diagnóstico foi de 149 (IC 95%: 18-1.243). A DWI teve uma AUC de 0,97 (IC 95%: 0,95-0,98).

**Conclusão:**

DWI é uma técnica reprodutível que demonstrou alta acurácia na diferenciação de estados malignos e benignos nos linfonodos torácicos.

## INTRODUCTION

A wide range of diseases are associated with thoracic lymphadenopathy^(1)^. Evaluating enlarged lymph nodes is clinically essential for treatment planning and for the prediction of the prognosis^(2)^. The main concern when evaluating a patient with a novel finding of enlarged thoracic lymph nodes, without a previous diagnosis, is the determination of whether the etiology is malignant or benign.

Several techniques and procedures are available to assess thoracic lymphadenopathy. Although biopsy is the recommended method for diagnosis, the risks associated with the procedure and sampling error have driven clinicians to search for noninvasive diagnostic methods^(3,4)^. Computed tomography (CT) is typically the technique of choice for thoracic assessment and for the morphological description of enlarged lymph nodes. Chest CT is frequently used in the investigation of patient complaints of respiratory symptoms, often revealing enlarged thoracic lymph nodes. However, CT cannot accurately differentiate benign from malignant lymph nodes and exposes patients to radiation^(5,6)^. Positron emission tomography/CT (PET/CT) performed with fluorine-18-fluorodeoxyglucose (^18^F-FDG) relies on the biochemical mechanism of increased glucose uptake by the malignant cells to differentiate malignant from benign lymph nodes, appearing to be better than is CT alone^(7)^. However, PET/CT is often unavailable and frequently provides false-positive results^(8,9)^. Diffusion-weighted magnetic resonance imaging (DWI) is a noninvasive, radiation-free tool that does not require the use of exogenous contrast agents, is accessible, and is easy to incorporate into the clinical routine. Over the last decade, DWI has demonstrated good diagnostic performance in multifarious tumors of various etiologies^(10-14)^. The aim of this study was to determine the performance of DWI in distinguishing between malignant and benign thoracic lymph nodes.

## MATERIALS AND METHODS

This meta-analysis was conducted in accordance with the criteria established in the Preferred Reporting Items for Systematic Reviews and Meta-Analyses (PRISMA) statement^(15)^. The International Prospective Register of Systematic Reviews was searched in order to identify unpublished systematic reviews. Because the study utilized anonymous data from previously published studies and thus presented no risk to the subjects, the institutional review board waived the requirement for written informed consent.

### Search strategy

We performed searches of the literature in the databases MEDLINE (via PubMed), EMBASE, and Web of Science, from their inception through April 2020. The search strategy included the use of the following terms and medical subject headings: “Magnetic Resonance Imaging” OR “MRI” OR “MR imaging” AND “mediastinum” OR “chest” OR “thorax” OR “thoracic” OR “hilar” AND “lymph nodes” OR “lymphadenopathy”. The strategies for other databases are available upon request. Articles published in English, Portuguese, and Spanish were included. We also performed manual searches of the references of the articles selected. Disagreements regarding the selection of articles were resolved by consensus.

### Eligibility criteria

The criteria for the inclusion of articles were the following: reporting results of magnetic resonance imaging (MRI) evaluation of thoracic lymph nodes; reporting apparent diffusion coefficient (ADC) measurements; and having used the histopathological analysis as the reference standard. Studies with missing or poor-quality data were excluded, as were those not involving DWI of the chest, those that were nondiagnostic studies, and those that were published as a conference abstract, letter, review, animal study, comment, or case report. The following focused questions were addressed: What is the applicability of DWI of thoracic lymph nodes in differentiating between malignant and benign lymph nodes in patients without a prior diagnosis?; What has been investigated regarding the application of DWI in thoracic lymph node evaluation?; What results were obtained by the researchers?

### Data extraction

Two of the authors, working independently, evaluated the titles and abstracts of the articles retrieved, applying the inclusion and exclusion criteria. Disagreements were resolved by consensus with the help of a third author. The same reviewers independently evaluated the full texts of the articles and made their selection in accordance with the eligibility criteria. Studies accepted for analysis were assessed in accordance with the PRISMA guidelines. The following data were collected: first author; year of publication; study design; country of patient recruitment; patient demographic characteristics (age and gender); reference standard (histopathological analysis of surgical resection/biopsy sample or radiological follow-up); technical details of the MRI scanner, MRI characteristics (ADC values and number of nodes assessed); final pathological confirmation of the nature of the lymphadenopathy; and the prevalence of malignant and benign lymph nodes.

### Study quality assessment

The Quality Assessment of Diagnostic Accuracy Studies 2 (QUADAS-2) tool^(16)^ was the instrument of choice to evaluate the risk of bias of each study. The QUADAS-2 tool facilitates the process of evaluating studies, on the basis of four main domains: patient selection; index test(s); reference standard; and flow and timing. Its result is presented in the form of a graph showing whether the risk of bias or inapplicability is “low”, “unclear”, or “high”.

### Statistical analysis

The pooled sensitivities and specificities, together with the respective 95% confidence intervals (95% CIs), were calculated by using random-effects analysis. The pooled positive likelihood ratio, negative likelihood ratio, and diagnostic odds ratio (DOR) were also obtained. Summary receiver operating characteristic curves were constructed, and the areas under the curve (AUCs) were obtained. To assume an approximate normal distribution, we used the distribution of logit-transformed sensitivity and specificity, as well as that of the natural logarithm of DOR^(17-19)^. A Deeks’ funnel plot was used in order to display any publication bias. All statistical analyses were performed with Stata software, v. 15.0 (Stata Corp., College Station, TX, USA).

## RESULTS

**Figure 1:**
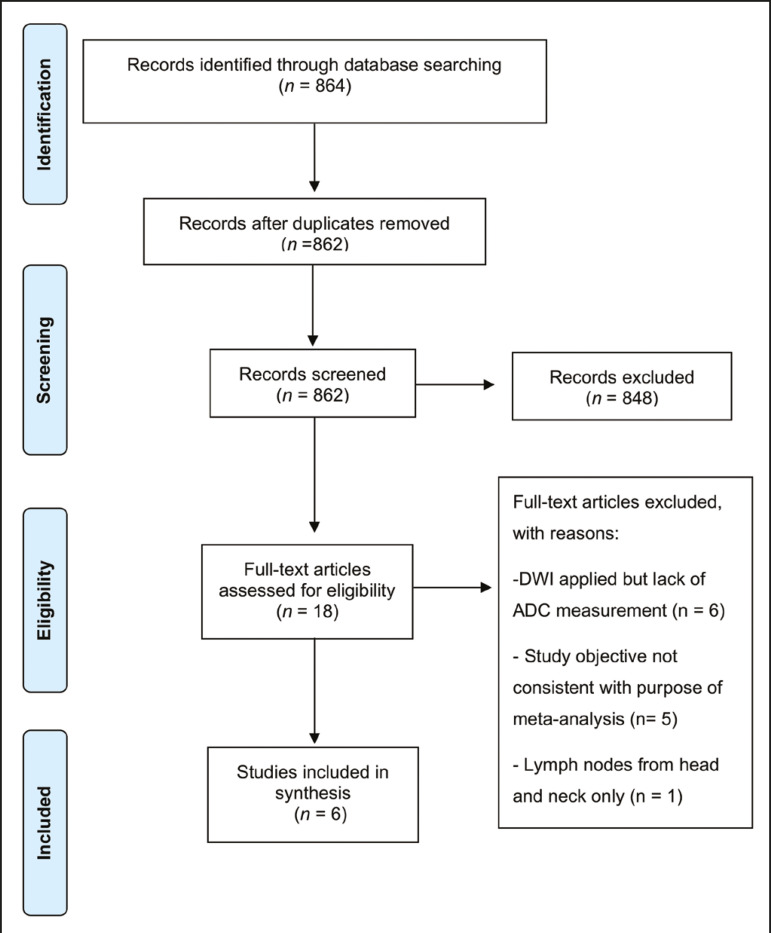
PRISMA flow chart detailing the search and article selection processes.

The search resulted in 864 potentially relevant citations from the electronic databases. After duplicate titles had been removed, 862 articles remained. During the screening of the titles and abstracts, another 848 articles were excluded. We evaluated the full texts of 18 remaining articles, 12 of which were thus excluded, yielding 6 articles that met our inclusion criteria^(20-25)^. Figure 1 depicts the article selection process.

**Figure 2:**
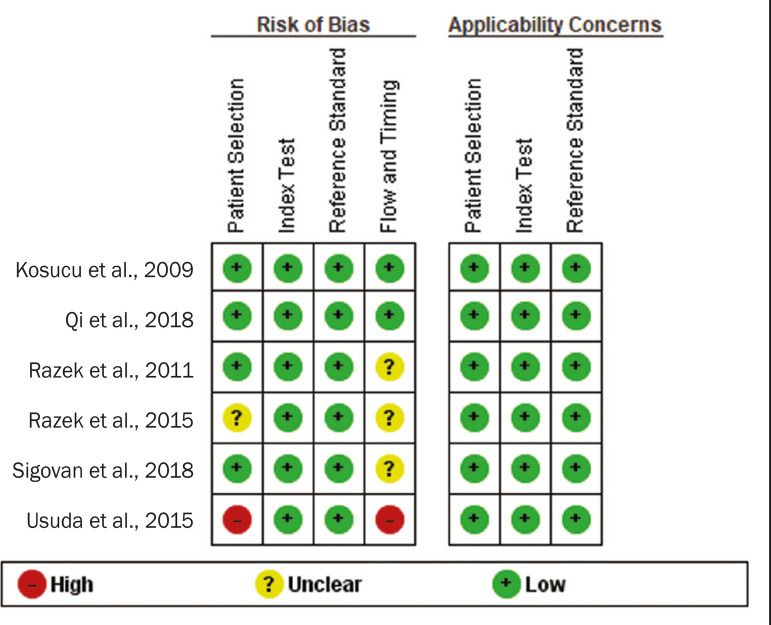
QUADAS-2 summary. The overall QUADAS-2 score for the articles selected suggests that they were of high quality.

Figure 2 shows the QUADAS-2 results. Although most of the included studies had a low risk of bias, the chance of possible confounders was greater in two domains: flow and timing; and patient selection. In four studies^(21-23,25)^, the authors do not mention the time between histological analysis and acquisition of the imaging test. In two studies^(8,23)^, patients with previously identified PET/CT alterations were selected.

The articles selected for review evaluated a collective total of 356 thoracic lymph nodes in 214 patients (Table 1). The underlying non-neoplastic pathologies, as described by the authors, ranged from sarcoidosis to reactive lymphoid hyperplasia, necrotizing granulomatous lymphadenitis, tuberculous nodes, pneumoconiosis/silicosis, Langerhans cell histiocytosis, and histoplasmosis. Malignant thoracic lymph nodes were metastases of small cell lung cancer, non-small cell lung cancer, or lymphoma, as well as metastatic lymph nodes from distant sites or leukemia. Two studies did not provided specified diagnosis per lymph node, justifying that choice by stating that there were no significant differences between the etiologies of the malignant lymph nodes and those of the benign lymph nodes^(8,25)^. The selected studies enrolled patients variously in Europe^(25)^, Asia^(20,23,24,26)^, and Africa^(21,22)^. Table 1 presents a summary of the studies.

**Table 1:** Summary of the studies selected.

Reference	Country	Study design	Number of patients	Total number of MLNs (benign MLNs)	Field strength	b-value (s/mm^2^)	Result			
							TP	FP	FN	TN
Kosucu et al.^(20)^	Turkey	Prospective	35	91 (72)	1.5 T	50; 400	19	0	0	72
Razek et al.^(22)^	Egypt	Prospective	35	35 (7)	1.5 T	0; 300; 600	27	2	1	5
Razek et al.^(21)^	Egypt	Retrospective	32	29 (9)	1.5 T	0; 300; 600	20	2	0	7
Usuda et al.^(23)^	Japan	Prospective	23	23 (16)	1.5 T	0; 800	5	0	2	16
Qi et al.^(24)^	China	Prospective	35	91 (49)	1.5 T	0; 50; 100; 200; 400; 600; 800; 1,000	32	5	10	44
Sigovan et al.^(25)^	France	Prospective	54	87 (65)	3.0 T	0; 400; 800	15	10	7	55

MLNs, mediastinal lymph nodes; TP, true-positive; FP, false-positive; FN, false-negative; TN, true-negative.

**Figure 3:**
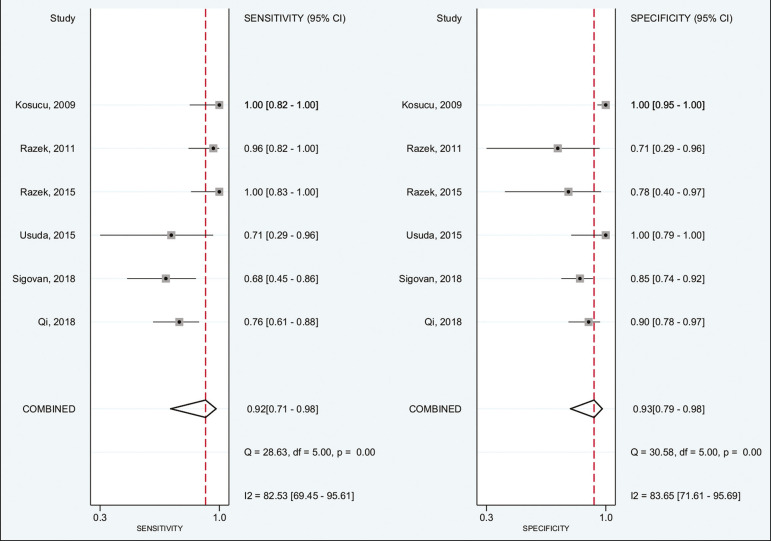
Forest plots of the sensitivity and specificity of DWI for differentiating between malignant and benign thoracic lymph nodes. Heterogeneity was high, as evidenced by the I2 value, which was 82%, values over 75% being considered indicative of high heterogeneity.

**Figure 4:**
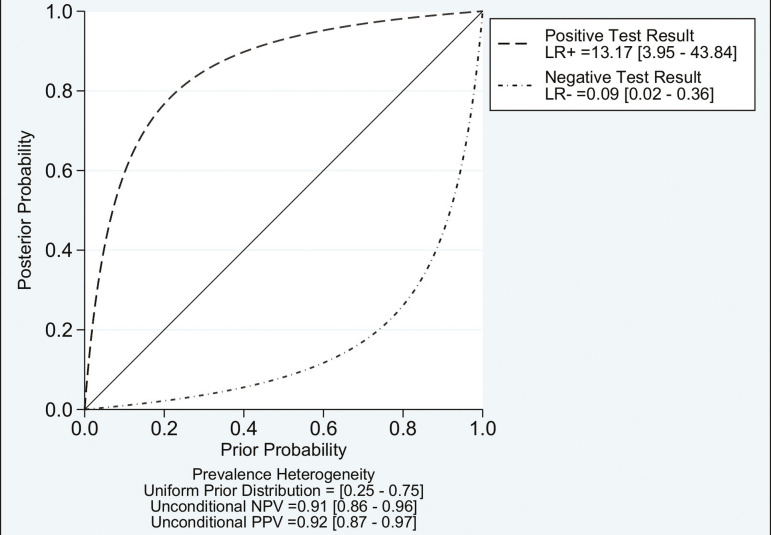
Conditional probability plot of the ability of DWI to differentiate between malignant and benign mediastinal lymph nodes. LR, likelihood ratio.

**Figure 5:**
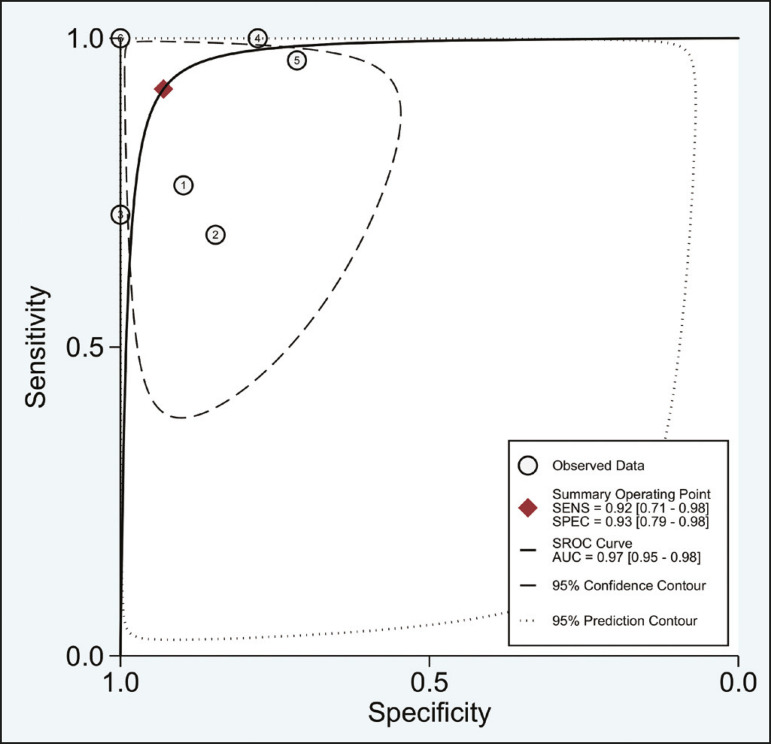
Receiver operating characteristic curve for DWI. AUC suggest a high diagnostic performance. Numbers within circles represent the studies referenced.

Of the 356 thoracic lymph nodes evaluated, 211 (59.2%) were found to be benign and 145 (40.8%) were found to be malignant. The pooled sensitivity and specificity of DWI was 92% (95% CI: 71-98%) and 93% (95% CI: 79-98%), respectively, as shown in Figure 3. The sensitivity and specificity of DWI showed strong heterogeneity (I^2^ = 82.5% and 83.6%, respectively). A forest plot of the DOR values was used in order to evaluate the determination of heterogeneity caused by a non-threshold effect. In the selected studies, heterogeneity was indicated by a nonlinear distribution of the DOR values. The positive and negative likelihood ratios were 13.2 (95% CI: 4.0-43.8) and 0.09 (95% CI: 0.02-0.36), respectively, as shown in Figure 4. The DOR was 149 (95% CI: 18-1,243). As can be seen in Figure 5, DWI had an AUC of 0.97 (95% CI: 0.95-0.98).

## DISCUSSION

Recent studies conducted in Brazil have highlighted the importance of CT in the evaluation of thoracic diseases^(27-34)^. However, the role of DWI has been little emphasized. DWI is a noninvasive technique that measures the water diffusion in biological tissues. The natural motion of water can be measured as signal loss and expressed as an ADC value. The ADC depends on the presence of obstacles to diffusion (Brownian motion), such as increased cellularity and macromolecules that can be found in neoplastic/malignant disease^(35,36)^. Lymph nodes affected by metastasis show lower ADC values than do benign tumors. Malignant tumors-due to enlarged nuclei, hyperchromatic cells, angulated nuclear contours, and hypercellularity-have reduced water diffusion in their extracellular and intracellular domains^(37,38)^. Thoracic analysis utilizing DWI has progressed in recent years. In comparison with CT, it has the advantages of not exposing patients to radiation, not requiring the administration of intravenous contrast, and short examination times. In comparison with ^18^F-FDG-PET/CT, the use of DWI has those same benefits, as well as being more widely available and producing fewer false-positive results^(8)^. When lymph nodes contain inflammation, ^18^F-FDG PET/CT is likely to show false-positive results, whereas it is likely to show false-negative results when lymph nodes contain small amounts of cancer cells^(39)^. All of the included studies reported results showing that DWI can be useful in differentiating between lymph nodes with metastasis and those without, with good accuracy in patients with enlarged thoracic lymph nodes. The studies also showed that the ADC values were significantly lower for benign lymph nodes than for malignant lymph nodes.

Whereas other studies on the use of DWI for thoracic lymph nodes have focused on the nodal stage assessment for patients with previously identified primary tumors, ours is the first study to systematically attempt to clarify the quantitative distinction between malignant and benign lymph nodes through the use of DWI in patients without an initial diagnosis^(40,41)^. This ability of DWI is relevant because it allows diseases that can manifest primarily in lymph nodes, such as lymphoma and sarcoidosis, to be differentiated. Previous articles have demonstrated that DWI and ^18^F-FDG-PET/CT both have good diagnostic performance in evaluating lymph nodes in non-small cell lung cancer. In the preoperative staging of mediastinal and hilar lymph nodes^(39)^, DWI has been found to show a specificity for N staging of 95%, significantly higher than the 89% observed for ^18^F-FDG-PET/CT^(39)^. One study^(23)^ reported that the specificity of DWI was 100%, suggesting that this modality can give fewer false-negative results for malignant lymph nodes, excluding mediastinal lymph node metastases. The authors of that study compared DWI and ^18^F-FDG-PET/CT in thoracic lymph nodes with ^18^F-FDG accumulation (maximum standardized uptake value > 3) in 33 patients, demonstrating that the ADC value was significantly lower in malignant than in benign mediastinal lymph nodes, although the maximum standardized uptake value did not differ significantly between the two. Sigovan et al.^(25)^ and Qi et al.^(24)^ demonstrated that DWI had a specificity superior than 80% for the diagnosis of malignant lymph nodes. Sigovan et al.^(25)^ tested the applicability of ADC mapping, as well as the relative contrast ratio between the signal intensity of a lesion (lymph node) and that of muscle, using a 3-T scanner, unlike the other five studies included, all of which employed 1.5-T scanners. The authors found no significant difference between the diagnostic accuracy of ADC mapping and that of the relative contrast ratio. Qi et al.^(24)^ investigated the value of an intravoxel incoherent motion diffusion model to the same purpose as the other studies, with the application of eight b-values (0, 50, 100, 200, 400, 600, 800, and 1,000 s/mm^2^), combining perfusion and diffusion in order to characterize tissues, whereas the use of a low b-value to calculate the ADC is subject to errors introduced by the microcirculation of blood. Those authors showed that the combination of ADC mapping and the microvascular volume fraction provides diagnostic performance better than that of ADC mapping (mono-exponential model DWI) alone for discriminating between malignant and benign thoracic lymph nodes. Kosucu et al.^(20)^ found that the use of a quantitative measure such as the ADC is more reliable than is the qualitative description of affected nodes, because malignant and benign lymph nodes can both show heterogeneous signal intensity. The authors reported that, of the malignant lymph nodes identified, 47.36% were hypointense on DWI and 73.68% were hypointense on ADC maps, compared with 80.55% and 79.16% of the benign lymph nodes, respectively. In 2011, Razek et al.^(22)^ analyzed the role of DWI in predicting malignant and benign mediastinal lymph nodes. In a subsequent study, conducted in 2015, the same research team compared ^18^F-FDG-PET/CT and DWI for the analysis of mediastinal lymphadenopathy in children (2-15 years of age)^(21)^. In the 2011 study, the authors found that DWI had an accuracy of DWI of 83.9%, a sensitivity of 96.4%, a specificity of 71.4%, a negative predictive value of 95.2%, and a positive predictive value of 77.1%, compared with 93.1%, 100%, 77.8%, 90.9%, and 100%, respectively, in the 2015 study, showing that the ADC map is a promising parameter that can help in the differentiation between malignant and benign lymph nodes, which is highly desirable in children, in whom the risks of radiation exposure are greater.

Cutoff values for ADC parameters were not the same in all of the studies included in our meta-analysis. This variation may be explained by the ADC values for lymph nodes due to the type of diffusion data acquisition and the b-values employed. Variations in b-values can change the diffusion sensitivity. The highest sensitivity values, of 96.4% and 100%, were seen in the previously cited studies conducted in 2011 and 2015, respectively^(21,22)^. The lowest sensitivity values, of 68.2% and 62.2%, were seen in the studies conducted by Sigovan et al.^(25)^ and Kosucu et al.^(20)^, respectively. That could be explained by the fact that malignant and benign lymph nodes of different pathologies were compared. However, the use of free-breathing DWI sequences can improve diagnostic performance for assessing mediastinal lymph nodes over conventional DWI in terms of the signal-to-background ratio, fat suppression, and the number of motion artifacts^(42)^. The modalities employed among the studies evaluated in our meta-analysis included cardiac gating^(24)^, respiratory-triggering^(20,23)^, and free-breathing^(21,25)^. Nevertheless, DWI has some limitations, such as the fact that benign lesions can exhibit restricted diffusion, thus mimicking malignant lesions^(43,44)^, abscesses, and thrombi^(45,46)^.

The DWI technique is quite useful, particularly for determining the status of mediastinal lymph nodes. It can reduce the frequency of unnecessary invasive procedures, yielding a less harmful diagnostic pathway. Although there is a need for further studies addressing interobserver reproducibility, as well as for cost-effectiveness analyses, we encourage the routine use of ADC mapping as an adjunct tool for the diagnosis of thoracic lymphadenopathy.

## References

[1] Mennini ML, Catalano C, Del Monte M (2012). Computed tomography and magnetic resonance imaging of the thoracic lymphatic system. Thorac Surg Clin.

[2] Passlick B (1999). Detection of disseminated lung cancer cells in lymph nodes by monoclonal antibodies: impact on staging and prognosis. Ann Ital Chir.

[3] Gupta S, Seaberg K, Wallace MJ (2005). Imaging-guided percutaneous biopsy of mediastinal lesions: different approaches and anatomic considerations. Radiographics.

[4] Goldberg SN, Raptopoulos V, Boiselle PM (2000). Mediastinal lymphadenopathy: diagnostic yield of transbronchial mediastinal lymph node biopsy with CT fluoroscopic guidance-initial experience. Radiology.

[5] Torabi M, Aquino SL, Harisinghani MG (2004). Current concepts in lymph node imaging. J Nucl Med.

[6] Arita T, Matsumoto T, Kuramitsu T (1996). Is it possible to differentiate malignant mediastinal nodes from benign nodes by size? Reevaluation by CT, transesophageal echocardiography, and nodal specimen. Chest.

[7] Steinert HC, Hauser M, Allemann F (1997). Non-small cell lung cancer: nodal staging with FDG PET versus CT with correlative lymph node mapping and sampling. Radiology.

[8] Usuda K, Sagawa M, Motono N (2013). Advantages of diffusion-weighted imaging over positron emission tomography-computed tomography in assessment of hilar and mediastinal lymph node in lung cancer. Ann Surg Oncol.

[9] Nomori H, Mori T, Ikeda K (2008). Diffusion-weighted magnetic resonance imaging can be used in place of positron emission tomography for N staging of non-small cell lung cancer with fewer false-positive results. J Thorac Cardiovasc Surg.

[10] Gilet AG, Kang SK, Kim D (2012). Advanced renal mass imaging: diffusion and perfusion MRI. Curr Urol Rep.

[11] Schaefer PW (2001). Applications of DWI in clinical neurology. J Neurol Sci..

[12] Mertan FV, Berman R, Szajek K (2016). Evaluating the role of mpMRI in prostate cancer assessment. Expert Rev Med Devices.

[13] Matoba M, Tonami H, Kondou T (2007). Lung carcinoma: diffusion-weighted MR imaging-preliminary evaluation with apparent diffusion coefficient. Radiology.

[14] Taouli B, Vilgrain V, Dumont E (2003). Evaluation of liver diffusion isotropy and characterization of focal hepatic lesions with two single-shot echo-planar MR imaging sequences: prospective study in 66 patients. Radiology.

[15] Moher D, Liberati A, Tetzlaff J (2009). Preferred reporting items for systematic reviews and meta-analyses: the PRISMA statement. PLoS Med.

[16] Whiting PF, Rutjes AWS, Westwood ME (2011). QUADAS-2: a revised tool for the quality assessment of diagnostic accuracy studies. Ann Intern Med.

[17] Hanley JA, McNeil BJ (1983). A method of comparing the areas under receiver operating characteristic curves derived from the same cases. Radiology.

[18] Kiewiet JJS, Leeuwenburgh MMN, Bipat S (2012). A systematic review and meta-analysis of diagnostic performance of imaging in acute cholecystitis. Radiology.

[19] Glas AS, Lijmer JG, Prins MH (2003). The diagnostic odds ratio: a single indicator of test performance. J Clin Epidemiol.

[20] Kosucu P, Tekinbas C, Erol M (2009). Mediastinal lymph nodes: assessment with diffusion-weighted MR imaging. J Magn Reson Imaging.

[21] Razek AAKA, Gaballa G, Elashry R (2015). Diffusion-weighted MR imaging of mediastinal lymphadenopathy in children. Jpn J Radiol.

[22] Razek AAKA, Elkammary S, Elmorsy AS (2011). Characterization of mediastinal lymphadenopathy with diffusion-weighted imaging. Magn Reson Imaging.

[23] Usuda K, Maeda S, Motono N (2015). Diagnostic performance of diffusion-weighted imaging for multiple hilar and mediastinal lymph nodes with FDG accumulation. Asian Pac J Cancer Prev.

[24] Qi LP, Yan WP, Chen KN (2018). Discrimination of malignant versus benign mediastinal lymph nodes using diffusion MRI with an IVIM model. Eur Radiol.

[25] Sigovan M, Akl P, Mesmann C (2018). Benign and malignant enlarged chest nodes staging by diffusion-weighted MRI: an alternative to mediastinoscopy?. Br J Radiol.

[26] Nakayama J, Miyasaka K, Omatsu T (2010). Metastases in mediastinal and hilar lymph nodes in patients with non-small cell lung cancer: quantitative assessment with diffusion-weighted magnetic resonance imaging and apparent diffusion coefficient. J Comput Assist Tomogr.

[27] Geraldino ACC, Marchiori E (2019). Cavitary rheumatoid nodules: an unusual pulmonary finding. Radiol Bras.

[28] Tibana TK, Camilo DMR, Nunes TF (2019). Congenital lobar emphysema. Radiol Bras.

[29] Santos RFT, Tibana TK, Adôrno IF (2019). Mounier-Kuhn syndrome: an unusual cause of bronchiectasis. Radiol Bras.

[30] Avelino EBP, Verza L, Neves T (2019). Lymphocytic interstitial pneumonia and pulmonar amyloidosis in Sjögren's syndrome. Radiol Bras.

[31] Barbosa PNVP, Bitencourt AGV, Miranda GD (2020). Chest CT accuracy in the diagnosis of SARS-CoV-2 infection: initial experience in a cancer center. Radiol Bras.

[32] Müller CIS, Müller NL (2020). Chest CT target sign in a couple with COVID-19 pneumonia. Radiol Bras.

[33] Louza GF, Nobre LF, Mançano AD (2020). Lymphocytic interstitial pneumonia: computed tomography findings in 36 patients. Radiol Bras.

[34] Farias LPG, Strabelli DG, Fonseca EKUN (2020). Thoracic tomographic manifestations in symptomatic respiratory patients with COVID-19. Radiol Bras.

[35] Koh DM, Collins DJ (2007). Diffusion-weighted MRI in the body: applications and challenges in oncology. AJR Am J Roentgenol.

[36] Chawla S, Kim S, Wang S (2009). Diffusion-weighted imaging in head and neck cancers. Future Oncol.

[37] Chen L, Liu M, Bao J (2013). The correlation between apparent diffusion coefficient and tumor cellularity in patients: a meta-analysis. PLoS One.

[38] Xu L, Tian J, Liu Y (2014). Accuracy of diffusion-weighted (DW) MRI with background signal suppression (MR-DWIBS) in diagnosis of mediastinal lymph node metastasis of nonsmall-cell lung cancer (NSCLC). J Magn Reson Imaging.

[39] Wu LM, Xu JR, Gu HY (2012). Preoperative mediastinal and hilar nodal staging with diffusion-weighted magnetic resonance imaging and fluorodeoxyglucose positron emission tomography/computed tomography in patients with non-small-cell lung cancer: which is better?. J Surg Res.

[40] Baxter GC, Graves MJ, Gilbert FJ (2019). A meta-analysis of the diagnostic performance of diffusion MRI for breast lesion characterization. Radiology.

[41] Chen L, Zhang J, Bao J (2013). Meta-analysis of diffusion-weighted MRI in the differential diagnosis of lung lesions. J Magn Reson Imaging.

[42] Mesmann C, Sigovan M, Berner LP (2014). Evaluation of image quality of DWIBS versus DWI sequences in thoracic MRI at 3T. Magn Reson Imaging.

[43] Humphries PD, Sebire NJ, Siegel MJ (2007). Tumors in pediatric patients at diffusion-weighted MR imaging: apparent diffusion coefficient and tumor cellularity. Radiology.

[44] Feuerlein S, Pauls S, Juchems MS (2009). Pitfalls in abdominal diffusion-weighted imaging: how predictive is restricted water diffusion for malignancy. AJR Am J Roentgenol.

[45] Desprechins B, Stadnik T, Koerts G (1999). Use of diffusion-weighted MR imaging in differential diagnosis between intracerebral necrotic tumors and cerebral abscesses. AJNR Am J Neuroradiol.

[46] Ohno Y, Niwa T, Nakanishi K (2010). Complementary roles of whole-body diffusion-weighted MRI and 18F-FDG PET: the state of the art and potential applications. J Nucl Med.

